# Modeling of Melt Flow and Heat Transfer in Stationary Gas Tungsten Arc Welding with Vertical and Tilted Torches

**DOI:** 10.3390/ma14226845

**Published:** 2021-11-12

**Authors:** Shahid Parvez, Md Irfanul Haque Siddiqui, Masood Ashraf Ali, Dan Dobrotă

**Affiliations:** 1 Mechanical Engineering Department, King Saud University, Riyadh 11421, Saudi Arabia; msiddiqui2.c@ksu.edu.sa; 2 Department of Industrial Engineering, College of Engineering, Prince Sattam Bin Abdulaziz University, Al-Kharj 16273, Saudi Arabia; mas.ali@psau.edu.sa; 3 Department of Industrial Engineering and Management, Faculty of Engineering, Lucian Blaga University of Sibiu, 550024 Sibiu, Romania

**Keywords:** melt flow in weld pool, gas tungsten arc welding, heat transfer to the filler wire, GTAW with filler wire, tilted torch, transient heat transfer in GTAW

## Abstract

A 3D numerical simulation was conducted to study the transient development of temperature distribution in stationary gas tungsten arc welding with filler wire. Heat transfer to the filler wire and the workpiece was investigated with vertical (90°) and titled (70°) torches. Heat flux, current flux, and gas drag force were calculated from the steady-state simulation of the arc. The temperature in the filler wire was determined at three different time intervals: 0.12 s, 0.24 s, and 0.36 s. The filler wire was assumed not to deform during this short time, and was therefore simulated as solid. The temperature in the workpiece was calculated at the same intervals using heat flux, current flux, gas drag force, Marangoni convection, and buoyancy. It should be noted that heat transfer to the filler wire was faster with the titled torch compared to the vertical torch. Heat flux to the workpiece was asymmetrical with both the vertical and tilted torches when the filler wire was fully inserted into the arc. It was found that the overall trends of temperature contours for both the arc and the workpiece were in good agreement. It was also observed that more heat was transferred to the filler wire with the 70° torch compared with the 90° torch. The melted volume of the filler wire (volume above 1750 °K) was 12 mm^3^ with the 70° torch, compared to 9.2 mm^3^ with the 90° torch.

## 1. Introduction

Gas tungsten arc welding (GTAW) is a joining method used widely in industry. A good deal of research is available to explore the process, either through experiments or computer simulations [1,2,3,4,5,6]. Since the process is highly transient, computer simulation is therefore helpful to understand its complete physics [7,8,9,10,11,12,13]. Various researchers have studied the plasma arc and the weld pool regions in steady or transient states, via computer simulations [14,15]. Substantial work is available in the study of heat transfer and fluid flow in gas metal arc welding (GMAW) with consumable wire [16,17,18,19,20,21,22], but minimal work can be found for GTAW.

Nemchinsky [23] studied heat transfer to the welding wire with the droplets in gas metal arc welding. The temperature in the consumable wire was calculated using the conduction equation. An analytical solution was developed by S. H. Kang and H. S. Cho [24] to determine the transient temperature distribution on a plate in GTAW with the addition of filler wire and with a 90° torch. A Gaussian heat source was considered to determine the temperature of the plate. A point heatsink was used to take away some of the heat at the location of the filler wire. Weld pool shapes were determined for various welding currents and wire feeding rates. Yudodibroto et al. [25] experimentally examined oscillations in the gas tungsten arc weld pool because of the addition of the filler wire. Four different metal feeding and melting methods (i.e., intermittent wire melting, uninterrupted bridging transfer, interrupted bridging transfer, and free flight transfer) were examined. It was concluded that uninterrupted metal melting did not significantly affect the weld pool oscillation signals. A mathematical model was developed by Chuan and Wu [26] to investigate the transient heat transfer and fluid flow in GTAW. The transient development of the weld pool was studied. Fan and Kovacevic [27] used the volume of fluid (VOF) method to simulate the melt depth and weld profiles transiently in GTAW with filler wire melting. The author used a Gaussian moving heat source for simplicity, although it was no more Gaussian with filler wire. It was observed that the rate of increase in the weld pool size decreased with time. The shape of the temperature contours in the leading part was found to be thinner than in the trailing part. Hybrid 2D/3D modeling of GTAW with the addition of filler wire was investigated by Traida et al. [1]. Heat flux, current flux, and arc pressure taken from the 2D simulation in [1] were applied to the workpiece in the 3D simulation in [28]. The filler wire was not specifically included in the arc plasma region, and the region was considered to be unaffected by the filler wire. The effect of the filler wire on the workpiece surface was modeled using the energy and momentum balance in the weld pool. An equation was used to consider the energy absorbed by the feeding filler wire and predict the weld pool shape. It was concluded the weld pool depth locally decreased in the area thermally affected by the filler wire. Chen et al. [29] explored arcing-wire GTAW experimentally; it was observed that the wire melting mechanism for arcing-wire GTAW is similar to that of gas metal arc welding (GMAW). A three-dimensional numerical simulation was performed by Parvez et al. [30] on a stationary arc to study the effect of torch angles in gas tungsten arc welding (GTAW) of SS304 stainless steel. A comparison was made to study the effects of 90° and 70° torch angles on the arc and the weld pool. Current density, heat flux, and gas shear stress were calculated in the arc region, and were used as inputs to the workpiece to determine the weld pool. It was found that for the 70° torch angle, the weld pool became asymmetrical, shallow, and wide ahead of the electrode tip in the welding direction. A mathematical model was developed by [31] to employ the energy, momentum, and mass transfer between the arc, filler wire, and workpiece domains; the droplet was also included in the simulation. The bead on the aluminum plate was studied; it was found that the peak arc temperature existed along the arc center near the anode. The larger the current, the hotter the arc along its axis, and the steeper the temperature gradient. The arc temperature was found to be maximal close to the anode, and minimal close to the cathode. It was also observed that the liquid molten pool in the central surface region of the molten pool flowed inward and downward, while the flow away from the weld center moved outward from high temperature to low temperature. This phenomenon was found to affect the weld pool size. Chen et al. [32] developed a 3D transient model to study the thermal and structural responses of steel plates in GTAW. Residual stress and deformation were studied. It was found that the convection coefficient becomes the temperature gradient in the cooling stage. The influence of the filler rod composition on the strength of magnesium alloy was studied in [33]. AZ31, AZ61, and AZ91 were used as filler materials in GTAW; it was concluded to use a filler rod so as not to exceed the solid solubility limit. Nomura et al. [34] experimentally studied the arc temperature in GTAW via spectroscopy, with a tilted torch; it was observed that the high-temperature area with a 30° tilted arc was larger compared with the vertical arc. A. B. Murphy [35] conducted a study on gas metal arc welding of aluminum plates and angled consumable wire; it was found that the orientation of the electrode wire strongly influenced the weld pool width and depth. A steeper angle led to a decreased reinforcement height and deeper penetration. Ishak and Salleh [36] studied the effects of different filler metals in metal inert gas (MIG) welding of aluminum alloy. Mechanical properties of the joint were investigated after welding with the filler metals ER4043 and ER5356. The grain size of the weldment was found to be smaller with ER4043 as compared to ER5356. The welding efficiency was high with the ER5356 electrode. The weldment with ER5356 was fractured at the heat-affected zone due to porosity, while with ER4043 it was fractured at the fusion zone due to the inclusion of oxides. 

A two-dimensional heat and mass transfer mathematical model was developed in [37]. The wire-based additive manufacturing process was considered with concentrated power sources. The model included equations for conjugate heat and mass transfer in free- surface liquid metal, including the differential equations of fluid motion, the Marangoni effect on the melt surface. Bead formation and temperature distribution were studied. Saheb and Chandrashekhar [38] experimentally studied the influence of the filler rod on the quality of the weld in GTAW. Mechanical tests were performed for the welds made with mild and stainless steel filler materials. It was concluded that for maximum strength, welding should be done using similar metals to achieve maximum solubility. Han et al. [39] experimentally examined the joining of AISI 1020 with AISI 304 using shielded metal arc welding and tungsten inert gas welding. The effect of filler material on joint strength was studied. Post-weld heat treatment was performed under temperatures of 600 °C, 630 °C, and 650 °C. It was concluded that TIG welding with MS filler rods and post-weld heat treatment at 600 °C are the optimal conditions for joining AISI 304 with AISI 1020. Khoraami et al. [40] studied the welding joint by using two dissimilar materials, i.e., plain carbon steel and AISI 430 ferritic stainless steel. Welding was conducted both in autogenous conditions and using ER309L austenitic filler rods, via the gas tungsten arc welding process. The results indicated that fully ferritic and duplex ferritic–martensitic microstructures were formed for autogenous and filler-added welds, respectively. Liang and Trelles [41] developed a novel coupling of plasma–electrode interaction and applied it to the 3D finite-volume simulation of a direct-current tungsten inert gas welding system. They reported that the region of the strongest electron overpopulation appeared at the intersection of the plasma fringes and the electrode surface. Some other relevant studies can be read elsewhere [42,43,44,45,46,47].

All previous research shows the arc behavior and its effect on the weld pool geometry by considering various process parameters. No study has been found to show the arc behavior with the insertion of filler rods and its consequences on the weld pool shape. In this paper, a more realistic TIG welding process is presented to examine heat transfer to the workpiece and the filler wire when it is fed to the arc plasma region. The simulation model is transient, and investigates the welding process with 90° and 70° torch angles. Heat flux to the filler wire and workpiece is determined. This heat flux will be used to simulate droplets’ formation, detachment, and impingement to the weld pool in a future study.

## 2. Numerical Details

The model was developed for stationary GTAW with feeding filler wire. The commercially available ANSYS CFX^®^ (version 16) software was used for simulation. The model can predict transient heat transfer to the filler wire and the workpiece with 90° and 70° torch angles. The welding parameters used in the simulation are given in Table 1. The arc temperatures are discussed at three different intervals to enable understanding of the physics in more detail. The arc plasma was simulated separately to determine heat flux to the filler wire and the workpiece in steady state. This heat flux was then used to calculate the temperature of the filler wire and the weld pool transiently.

Temperature-dependent material properties for the argon and SS304 steel are taken from [48,49,50]. The properties of the filler wire are assumed to be similar to those of SS304.

## 3. Governing Equations

The three-dimensional governing equations described in this section are taken from [51,52]. These equations are reproduced in order to explain the boundary conditions mentioned in Table 2 and Table 3.

The following assumptions are made for this study:

- The arc is steady and in LTE;
- The flow is turbulent in the arc and laminar in the weld pool region;
- The length of the tungsten electrode tip surrounded by the arc plasma is the same for the 90° and 70° torch angles;
- The filler wire and the workpiece material are assumed to have similar properties;
- The simulation time is short enough that the filler wire does not change its shape and remains solid. The same approach was followed as described in [52,53] for simplicity.

### 3.1. Governing Equations of the Arc Plasma

The arc is in a steady state, the flow is turbulent and non-buoyant, and there exist electric and magnetic fields. With these assumptions, the governing equations are:

Conservation of mass:

(1) ∇.(ρU)=0

The momentum equation:

(2) $$\nabla .\left(\rho U⊗U\right)-\nabla .\left(\mu_{eff}\nabla U\right)=\nabla p^{\prime }+\nabla .{\left(\mu_{eff}\nabla U\right)}^{T}+S_{M\;emag}$$

The energy equation:

(3) $$\nabla .\left(\rho Uh_{total}\right)=\nabla .\left(\kappa \nabla T\right)+S_{E\;emag}$$

The modified pressure $p^{\prime }$ in Equation (2) is calculated as:

(4) $$p^{\prime }=p+\frac{2}{3}\rho k+\frac{2}{3}\mu_{eff}\nabla .U$$

$\mu_{eff}$ in Equations (2) and (4) is given by:

(5) $$\mu_{eff}=\mu +\mu_{t}$$

$\mu_{t}$ in Equation (5) is determined as:

(6) $$\mu_{t}=C_{\mu }\rho \frac{k^{2}}{\varepsilon }$$

k and ε in Equation (6) are calculated as:

(7) $$\nabla .\left(\rho Uk\right)-\nabla .\left(\frac{\mu_{eff}}{\sigma_{k}}\nabla k\right)=P_{k}-\rho \varepsilon$$

(8) $$\nabla .\left(\rho U\varepsilon \right)-\nabla .\left(\frac{\mu_{eff}}{\sigma_{\varepsilon }}\nabla \varepsilon \right)=\frac{\varepsilon }{\kappa }\left(C_{1}P_{k}-C_{2}\rho \varepsilon \right)$$

$P_{k}$ in Equations (7) and (8) represents the production of k, and is determined as:

(9) $$P_{k}=\mu_{t}\nabla U.\left(\nabla U+{\left(\nabla U\right)}^{T}\right)-\frac{2}{3}\nabla .U\left(\mu_{t}\nabla .U+\rho k\right)$$

$S_{M\;emag}$ in Equation (2) is the Lorentz force, and is calculated as:

(10) $$S_{M\;emag}=J\times B$$

$S_{E\;emag}$ in Equation (3) represents Joule heating, and is determined as:

(11) $$S_{E\;emag}=J.B$$

*J* in Equations (10) and (11) is the current density, and is calculated as:

(12) J=σ(E+U×B)

And by Ohm’s law, *E* in Equation (12) is the electric field, and can be written as:

(13) $$E=\frac{J_{b}}{\sigma }$$

where *J_b_* is the current density at the electrode.

*B* in Equations (10)–(12) is the magnetic field, and is given by:

(14) B=∇×A

### 3.2. Governing Equations of the Filler Wire

In this study, only transient heat transfer to the filler wire is considered. The governing equation is:

(15) $$\frac{\partial \left(\rho h_{total}\right)}{\partial t}+\nabla .\left(\rho Uh_{total}\right)=\nabla .\left(\kappa \nabla T\right)$$

### 3.3. Governing Equations of the Weld Pool

The weld pool formation is transient, the flow is laminar and buoyant, and there are electric and magnetic fields. The modified governing equations are:

Conservation of mass:

(16) $$\frac{\partial \rho }{\partial t}+\nabla .\left(\rho U\right)=0\;$$

The momentum equation:

(17) $$\frac{\partial \left(\rho U\right)}{\partial t}+\nabla .\left(\rho U⊗U\right)-\nabla .\left(\mu \nabla U\right)=\nabla p+\nabla .{\left(\mu \nabla U\right)}^{T}+S_{M\;Bouy.}+S_{M\;emag}\;$$

The energy equation:

(18) $$\frac{\partial \left(\rho h_{total}\right)}{\partial t}+\nabla .\left(\rho Uh_{total}\right)=\nabla .\left(\kappa \nabla T\right)+S_{E\;emag}\;$$

*S_M Bouy._* is calculated using the Boussinesq approximation:

(19) $$S_{M\;Bouy.}=-\rho_{ref}\beta \left(T-T_{ref}\right)\;$$

## 4. Simulation Models

### 4.1. Model of the Arc Plasma

The 90° and 70° torch angles were considered in order to examine the transient heat transfer to the filler wire and the workpiece. The arc and weld pool behavior were studied as the filler wire fed into the plasma arc region.

Argon ionizes at a temperature higher than 7000 K; the arc is therefore initiated by introducing a heat source of 12,000 K. This heat source is then switched off once the arc is developed. The flow is steady, non-buoyant, and turbulent; the κ−ε model is used to characterize the arc flow. This turbulent model has been widely used in other, similar studies recently. This model introduces an effective viscosity in the momentum equation, which affects the velocity distribution in the arc column. The velocity distribution in the arc column using the κ−ε model has been compared in some previously published papers, which can be found elsewhere [54,55]. The magnetohydrodynamics (MHD) model is used to calculate the current density on the workpiece and filler wire surfaces. This current density is important in determining heat transfer to the filler wire and workpiece, and consequently decides the droplet and weld pool shapes.

Cathode and anode boundary layers are extremely important in calculating the behavior of the arc and the weld pool. These layers are also called the sheath regions, and are in the length of microns. The sheath region near the workpiece surface was modeled by employing a simplified method from [56], in which the heat transfer due to electron contribution is modeled according to Equation (20).

More than 40% of heat is transferred to the workpiece by the electron contribution [56]; this is modeled in physics terms by using Equation (20):

(20) $$F_{e}=J\left(\emptyset_{w}+V_{a}+V_{th}\right)+F_{r}\;$$

where $j\emptyset_{w}$ is the work function or potential energy given up by the electron upon entering the metal surface, $jV_{th}$ is the thermal energy carried by the electron from hot plasma to the cooler anode surface, and $jV_{a}$ is the kinetic energy acquired by the electron when it travels in the anode fall region. The anode fall is a transition region between the arc and the workpiece, where a sharp reduction in temperature occurs. The radiation effect (*F_r_*) was only 1.2%, and is therefore neglected. The values of $\emptyset_{w}$, $V_{a}$, and $V_{th}$ are 4.4 V, 1 V, and 2 V, respectively, as described in [56]. Net heat flux to the workpiece is caused by the electron contribution (*F_e_*), convective heat flux (*F_c_*), conductive heat flux, radiation, and metal vaporization. The effect of the conductive heat flux, radiation, and metal evaporation was less than 10% [56], and is therefore ignored in this study. Total heat flux on the filler wire and workpiece surfaces is calculated using Equation (21):

(21) $$F_{T}=F_{e}+F_{c}\;$$

**Figure 1:**
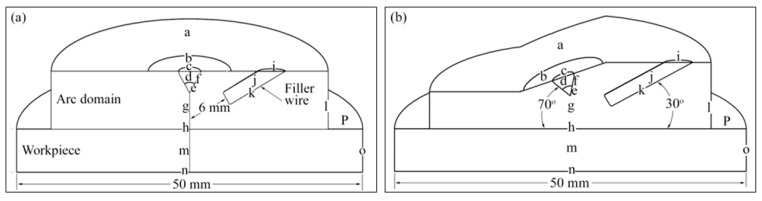
Computational domain: (**a**) 90°; (**b**) 70°.

The temperature of the tungsten electrode cannot go higher than 3000 K; therefore, it was set to this fixed temperature. A 200 A welding current was applied at the surface of the tungsten electrode (boundary *c* in Figure 1). The rest of the boundary conditions are mentioned in Table 2.

### 4.2. Model of Heat Transfer to the Filler Wire

The 2 mm diameter filler wire feeds into the arc with a velocity of 100 cm/min. The wire diameter and the feed velocity are taken from [1]. The simulation time of 0.36 s was chosen based on the 6.0 mm distance of the filler wire from the arc center, as shown in Figure 1a. This distance was the same for the vertical and tilted torches. It was assumed that this time would be short enough for the filler wire to remain non-deformed. After this time, the molten part of the filler wire deforms considerably and changes to a drop. The formation of the drop, its detachment from the filler wire, attachment to the workpiece, shape, and effect on the weld pool shape are beyond the scope of this work.

Heat flux given by Equation (21) was applied on the filler wire surface (boundary *K* in Figure 1), and simulation was performed for 0.12 s. At this time, the filler wire was 4 mm away from the arc center. For the second run, the physics were initialized with the previous time (at 0.12 s), and the simulation was continued for 0.24 s. The filler wire was now 2 mm from the arc center. The same procedure was adopted for the last timestep of 0.36 s. Boundary conditions are listed in Table 3 and Table 4.

### 4.3. Model of the Weld Pool

The liquid weld pool was simulated by defining the SS304 as fluid. A high viscosity of $1\times 10^{5}\;\mathrm{cp}$ was defined (in the material properties) where the temperature was less than 1750 K. Actual viscosity is defined where the temperature is higher. Higher viscosity assures zero convection, and those regions are considered solid.

The shape of the weld pool (width and depth) is determined by the four driving forces i.e., heat flux, current flux, gas drag force, Marangoni convection, and buoyancy. Heat flux, current flux, and gas drag force were taken from the steady-state results of the arc. The Boussinesq approximation was used for buoyancy calculation in the weld pool. Marangoni convection was calculated using Equation (22), taken from [57]:

(22) $$M_{a}=\frac{\partial \gamma }{\partial T}\left(\nabla T\right)\;$$

$\frac{\partial \gamma }{\partial T}$ in Equation (22) represents the surface tension gradient, and its values are taken from [58]. All four driving forces were applied to the workpiece surface, as mentioned in the boundary conditions in Table 4. The weld pool was determined transiently at 0.12 s, 0.24 s, and 0.36 s, with 90° and 70° torch angles.

### 4.4. Boundary Conditions

The details of boundary conditions applied to the arc and torch domains are shown in Table 2. Furthermore, Table 3 and Table 4 shows the details of boundary conditions applied to the filler wire and workpiece domains. Boundaries *a*–*p* shown in Figure 1 are explained in Table 2. In addition to this, Table 3 and Table 4 represent the boundary surfaces shown in Figure 1.

## 5. Model Validation

**Figure 2:**
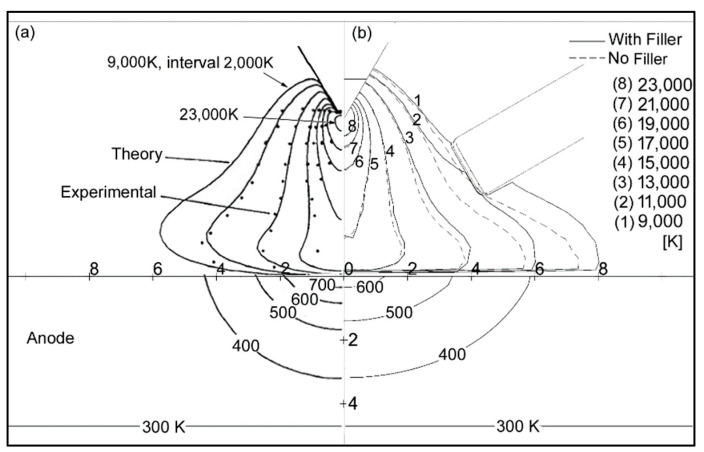
Temperature contours: (**a**) Lowke et al.; (**b**) present study.

The simulation model was validated with the previously available work of different authors. Figure 2 shows the temperature contours of the present study versus the previous work of Lowke et al. [59]. A welding current of 200 A, arc length of 5 mm, tip angle of 60°, and copper workpiece material were used for the latter study. The arc temperature contours in Figure 2b are shown with and without the filler wire. The filler wire is 6 mm away from the arc center. The contour of 9000 K in the arc region is found to be slightly wider due to the filler wire. The workpiece temperature contours are also observed to be wider because of the filler wire. The overall trends of temperature contours for both the arc and the workpiece are in good agreement.

**Figure 3:**
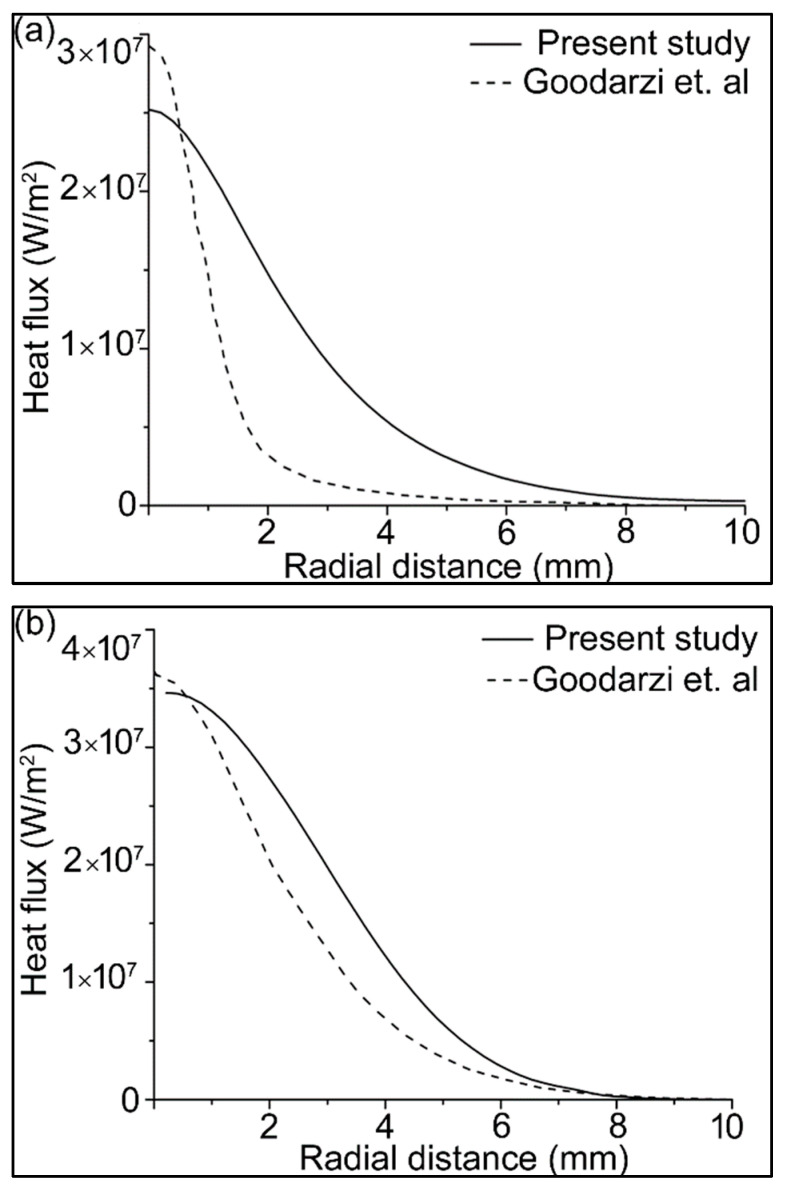

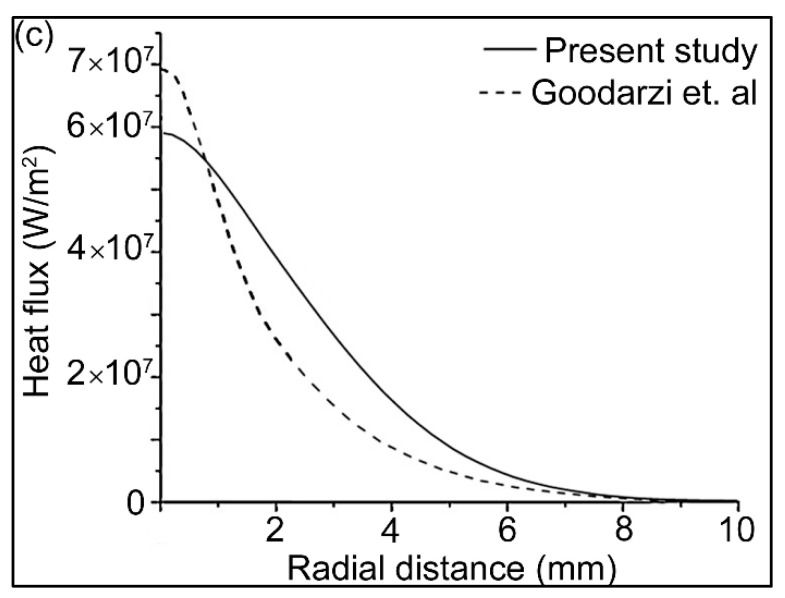
Heat flux on the workpiece surface due to (**a**) convection; (**b**) electron contribution; and (**c**) total heat flux.

Heat transfers to the workpiece because of the convective and electron heat fluxes, according to Equation (21). These heat fluxes are important for predicting an accurate weld pool. The heat fluxes were validated with the results of Goodarzi et al. [58], as shown in Figure 3. These results are in the absence of the filler wire, with the following welding parameters: current 200 A, arc length 5 mm, tip angle 60°.

There is a slight difference in the results, because the authors of [58] assumed laminar arc flow, while the arc flow considered in this study was turbulent. However, the results were found to be consistent with the experimental weld pool shapes.

**Figure 4:**
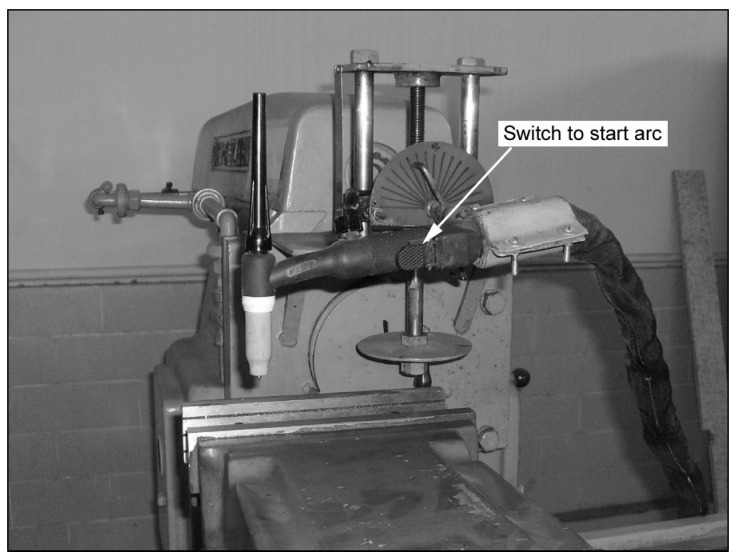
Testbed used in the experiments.

**Figure 5:**
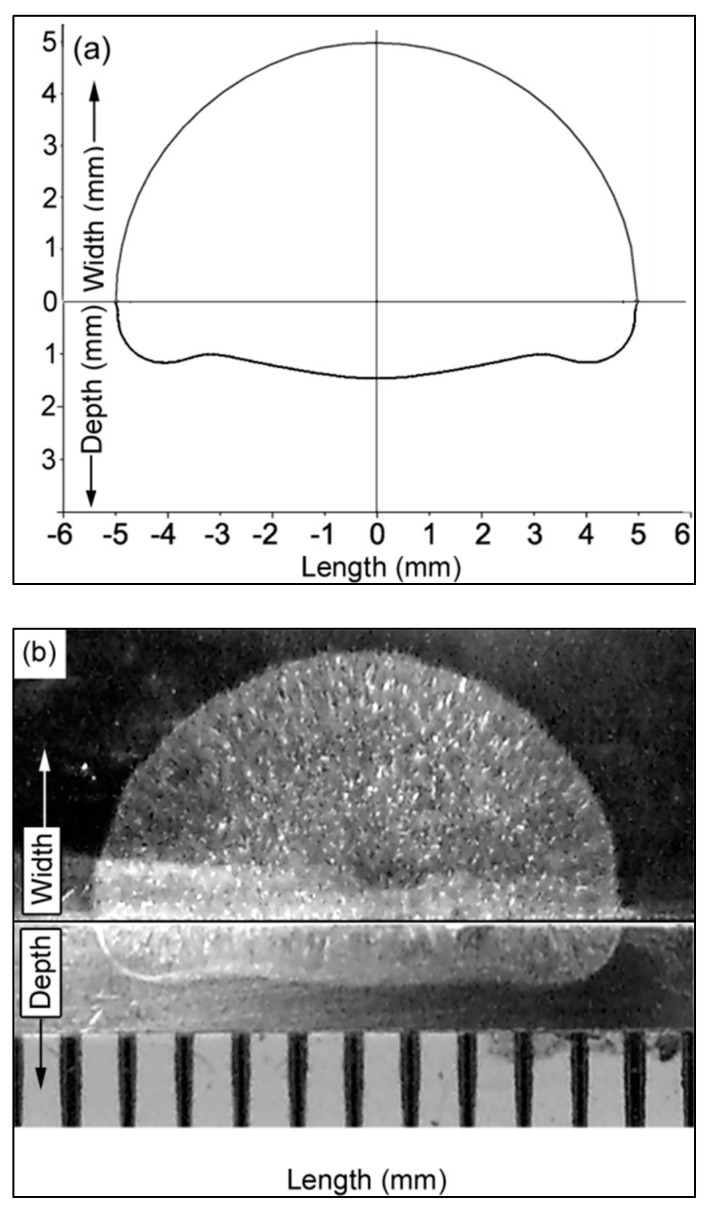
Weld pool shape: (**a**) numerical; (**b**) experimental.

The weld pool shape was determined numerically and experimentally after 2 s without the filler wire. In the experiments, the arc was DCEN (direct current electrode negative), and the welding current was 200 A. The shielding gas was argon, with a 14 L/min flow rate. The electrode was thoriated tungsten, 3.2 mm in diameter. The tip angle was 60°, and the arc length was 5 mm. The workpiece material was SS304, 50 mm in diameter and 10 mm thick. A Miller XMT 456 CC/CV multipurpose welding machine was used for the experiments. The switch in the welding torch shown in Figure 4 is connected to the timer, which ignites the arc after pressing. The timer switches off the arc automatically after two seconds. The numerical and experimental weld pool shapes shown in Figure 5 show good agreement and, therefore, validate the simulation model. The same simulation model was used to study the behavior of the arc and the weld pool transiently, with the addition of filler wire.

## 6. Results and Discussion

### 6.1. Temperatures in the Arc, Filler Wire, and Workpiece

**Figure 6:**
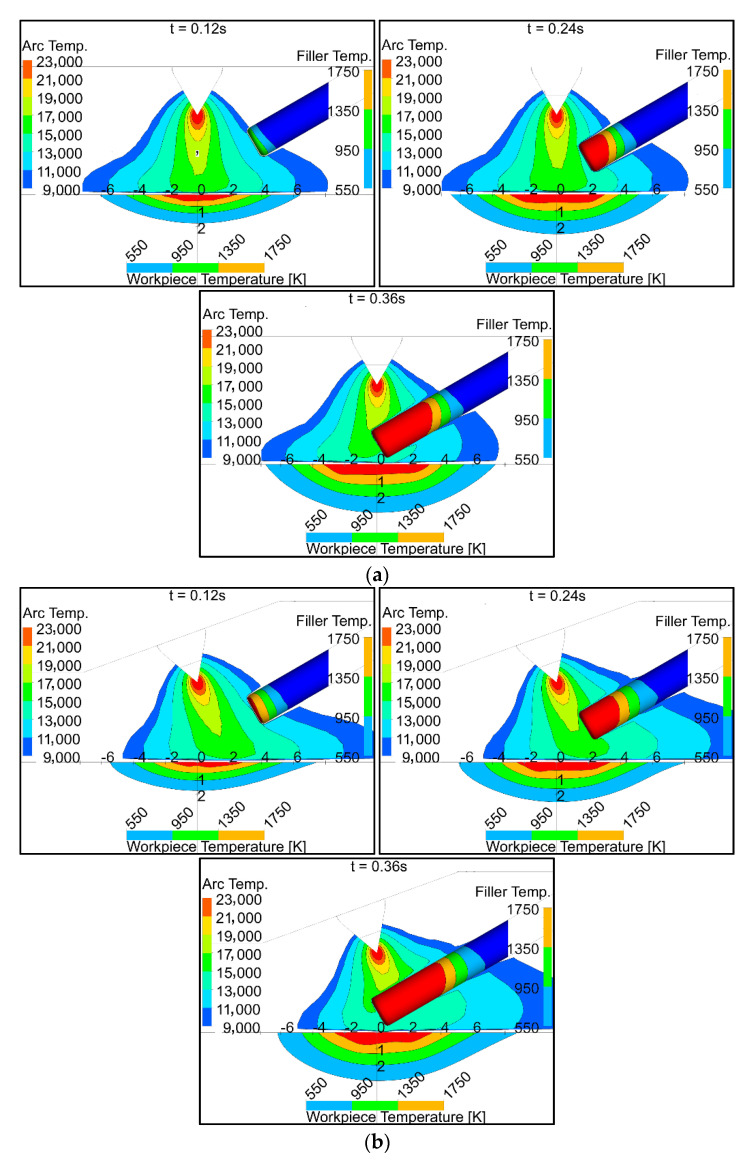
Temperature distribution in the arc, filler wire, and workpiece: (**a**) 90° torch; (**b**) 70° torch.

Temperature contours for three different intervals are shown in Figure 6. The red region near the electrode tip represents the plasma arc temperature, which is above 23,000 K. The red regions in the filler wire and the workpiece represent the molten part, where the temperature is above 1750 K. The filler wire moves towards the arc center with a velocity of 100 cm/min. More heat is transferred to the filler wire with the 70° torch compared to the 90° torch. The melted volume of the filler wire (volume above 1750 °K) was 12 mm^3^ with the 70° torch as compared to 9.2 mm^3^ with the 90° torch. The weld pool depth was symmetrical with the 90° torch when the filler wire was not fully inserted into the arc (i.e., at times 0.12 s and 0.24 s). The weld pool depth became non-symmetrical with the 90° torch when the filler wire was fully inserted into the arc (i.e., at time 0.36 s), because some heat was taken by the filler wire. With the 70° torch, the pool depth was consistently asymmetrical. The weld pool was slightly deeper and narrower with the 70° torch compared to the 90° torch, because more of the tungsten electrode tip area was exposed to the workpiece, and more electrons flowed towards the workpiece surface.

### 6.2. Convective Heat Flux to the Filler Wire

**Figure 7:**
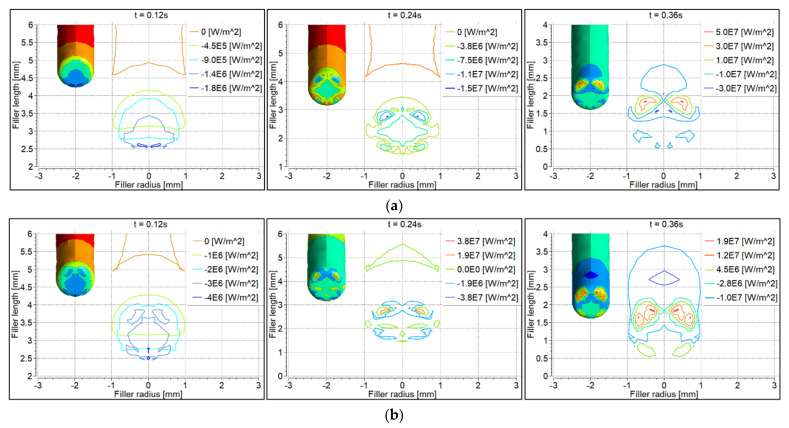
Convective heat flux distribution on the filler wire surface: (**a**) 90° torch; (**b**) 70° torch.

Convective heat flux to the feeding filler wire surface was determined transiently, as shown in Figure 7. Negative values of heat flux represent almost zero convective heat flux to the filler due to the high velocity of argon flow over the surface. At 0.12 s, convective heat flux to the filler wire was almost zero. At 0.24 s, heat flux was higher with the 70° torch compared to the 90° torch, because the filler wire was more directed towards the electrode tip (Figure 6a). The opposite was the case when more of the wire was fed after 0.36 s; the heat flux, therefore, was higher with the 90° torch compared to the 70° torch (Figure 6b).

### 6.3. Electron Heat Flux to the Filler Wire

**Figure 8:**
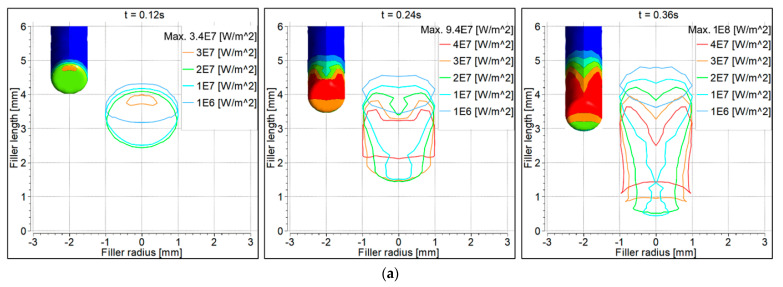

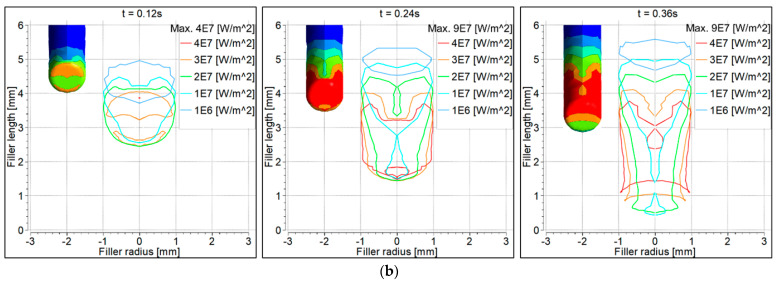
Electron heat flux distribution on the filler wire surface: (**a**) 90° torch; (**b**) 70° torch.

Electron heat flux was calculated according to Equation (20), as shown in Figure 8. Heat flux due to electron contribution was almost the same at intervals 0.12 s and 0.24 s. The heat flux was higher with the 90° torch, because more of the electrode tip area was exposed to the filler wire, compared to the 70° torch (Figure 6).

### 6.4. Total Heat Flux to the Filler Wire

Total heat flux was determined according to Equation (21) (see Figure 7 and Figure 8). Electron heat flux was much higher than convective heat flux. The distribution of the total heat flux was therefore the same as shown in Figure 8. However, it was observed that the distribution of the total heat flux was wider with the 70° torch compared to the 90° torch, which is why the filler wire started melting faster with the 70° torch compared to the 90° torch. This is also evident from Figure 6.

### 6.5. Convective Heat Flux to the Workpiece

**Figure 9:**
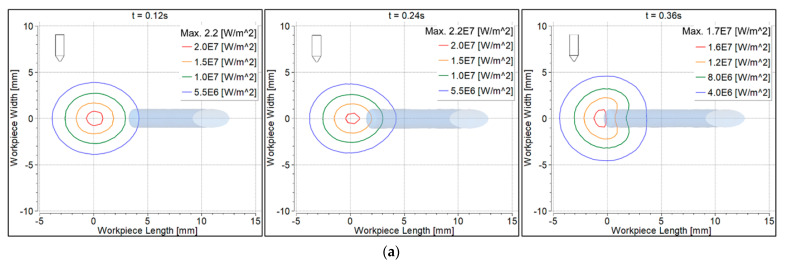

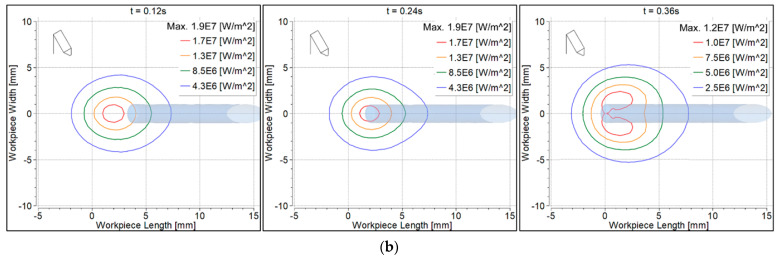
Convective heat flux distribution on the workpiece surface: (**a**) 90° torch; (**b**) 70° torch.

Convective heat flux to the workpiece surface is shown in Figure 9. At 0.12 s, the heat flux was symmetrical with the 90° torch, becoming non-symmetrical at 0.24 s and 0.36 s (Figure 9a). The heat flux was non-symmetrical with the 70° torch at all time intervals (Figure 9b). Heat flux beneath the filler wire was contracted at 0.36 s because some heat was taken by the filler wire.

### 6.6. Electron Heat Flux to the Workpiece

**Figure 10:**
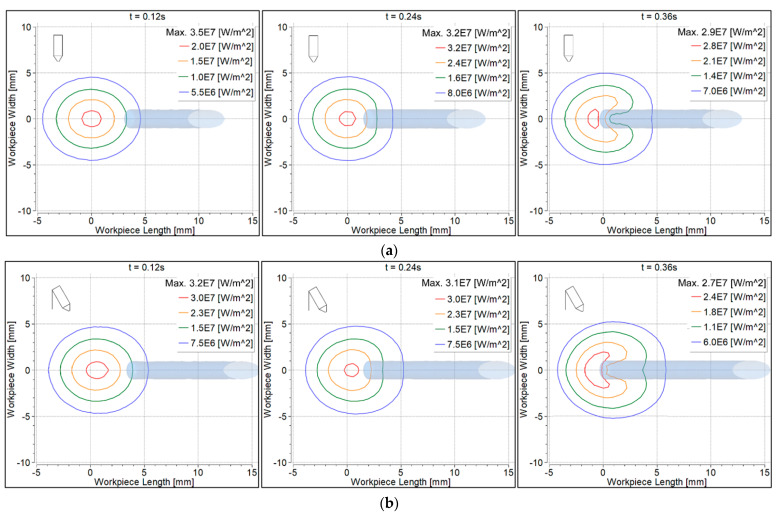
Electron heat flux distribution on the workpiece surface: (**a**) 90° torch; (**b**) 70° torch.

Figure 10 shows heat flux to the workpiece surface due to the electron contribution. The filler wire is shown as shaded. Heat flux distribution behavior was the same as discussed for the convective heat flux. Since the heat was taken by the filler wire, it was therefore contracted on the workpiece surface underneath the filler wire.

### 6.7. Total Heat Flux to the Workpiece

Total heat flux is the sum of the convective and electron heat fluxes. As discussed previously, heat flux due to electron contribution was higher than the convective heat flux. The graphs are very similar to those in Figure 10, and are therefore not shown. It is clear that when the filler wire was fully inserted, total heat flux on the workpiece surface was contracted below the filler wire.

### 6.8. The Weld Pool Shapes

**Figure 11:**
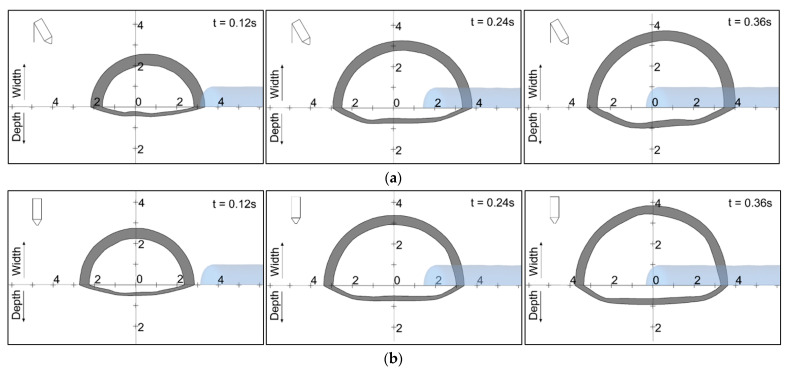
Weld pool width and depth: (**a**) 90° torch; (**b**) 70° torch.

The weld pool depth is presented in Figure 11 in more detail. The gray portion represents the mushy zone. The internal and external temperatures were 1750 °K and 1550 °K, respectively. The feeding filler wire is shown in blue, looking from the top of the workpiece. The development of the weld pool is shown transiently at three intervals. The width changed from nearly 4.2 mm (at 0.12 s) to 7 mm (at 0.36 s) with both the 90° and 70° torches. The width was observed to be symmetrical with the 90° torch, as shown in Figure 11a. The pool width was non-symmetrical with the 70° torch, and was away from the arc center in the direction of the torch, as shown in Figure 11b. Similar behavior was observed in [30], without the addition of filler wire.

## 7. Conclusions

The gas tungsten arc welding was simulated transiently with feeding filler wire. The results are presented at three intervals. The arc region was simulated separately in steady state in order to determine temperatures and heat flux in the filler wire and the workpiece. The transient temperature increase of the filler wire was studied by applying the total heat flux only. The filler wire was assumed not to deform in the given simulation time, and was treated as solid. The transient development of the weld pool was studied by applying the total heat flux, gas drag, Marangoni, and buoyancy forces on the workpiece. Heat flux to the filler wire was wider with the tilted torch compared to the vertical torch; the filler wire therefore started to melt earlier in the former case. Heat flux to the workpiece was non-symmetrical with the tilted torch. Heat flux to the workpiece was symmetrical with the vertical torch, and became nonsymmetrical when the filler wire was fed into it. The heat flux became contracted underneath the filler wire on the workpiece surface. The weld pool was deeper and narrower with the tilted torch compared to the vertical torch with the filler wire in. Whatever the torch angle, the weld pool shape was asymmetrical when the filler wire was fed into the workpiece. The simulation model heat fluxes were validated against the results of Goodarzi et al. [58]. It was noted that the overall trend of temperature contours for both the arc and the workpiece were in good agreement. It was observed that more heat was transferred to the filler wire with the 70° torch compared to the 90° torch. The melted volume of the filler wire (volume above 1750 °K) was 12 mm^3^ with the 70° torch compared to 9.2 mm^3^ with the 90° torch. Furthermore, it was found that with the 70° torch, the pool depth was consistently asymmetrical, and the weld pool was slightly deeper and narrower with the 70° torch compared to the 90° torch. Hence, more of the tungsten electrode tip area was exposed to the workpiece, so more electron flow was directed towards the workpiece surface. In this work, convective transient heat flux to the feeding filler wire surface was calculated. It was noted that, at 0.12 s, convective heat flux to the filler wire was almost zero. Electron heat flux was also calculated according to Equation (20), and it was well depicted and compared. Heat flux due to electron contribution was almost the same at intervals 0.12 s and 0.24 s. The weld pool depth and mushy zone were determined in simulation. The internal and external temperatures of the weld pool reached 1750 °K and 1550 °K, respectively. The width of the weld pool changed from nearly 4.2 mm (at 0.12 s) to 7 mm (at 0.36 s) with both the 90° and 70° torches.

## Figures and Tables

**Table 1:** Welding parameters used in the study.

Parameters	Value
Arc length	5 mm
Welding current	200 A
Argon flow	14 lt/min
Torch angle	90° and 70°
Tip angle	60°
Electrode diameter	3.2 mm
Filler wire diameter	2 mm
Filler wire angle	30°
Filler wire feed rate	100 cm/min

**Table 2:** Boundary details shown in Figure 1.

Boundary	Description
a	Top of the arc domain
b	Nozzle opening
c	Tungsten electrode cross-section
d	Tungsten electrode front symmetry plane
e	Tungsten electrode tip surrounded by the plasma
f	Arc–electrode interface
g	Arc front symmetry plane
h	Arc–workpiece interface
i	Filler rod cross-section
j	Filler rod front symmetry plane
k	Arc–filler rod interface
l	Outer of the arc domain
m	Workpiece front symmetry plane
n	Bottom of the workpiece
o	Exterior of the workpiece
p	Top of the workpiece

**Table 3:** Boundary conditions applied to the arc and torch domains.

			Arc Domain (Fluid)				Torch Domain (Solid)			
			Opening	Inlet	Sym.	Opening	Wall	Sym.	Interface	Interface
Equation No.	Boundary Variable	Units	*a*	*b*	*g*	*l*	*c*	*d*	*e*	*f*
(1) and (2)	*U*	m/s	-	$\frac{2.33\times 10^{-4}}{\pi r_{noz}^{2}}$	$U_{n}=0$	-	-	-	-	-
	*p*	Pa	$101.3\times 10^{3}$	-	-	$101.3\times 10^{3}$	-	-	-	-
(3)	*T*	K	303	303	$\frac{\partial T}{\partial n}=0$	303	3000	$\frac{\partial T}{\partial n}=0$	-	-
	*q*	W/m^2^	-	-	-	-	-	-	0	0
(12) and (13)	*E*	V/m	0	0	-	0	-	constant	$E_{side1}=E_{side2}$	0
	*V*	Volt	-	-	$\frac{\partial V}{\partial n}=0$	-	-	-	-	-
	$J_{b}$	A/m^2^	-	-	-	-	200πrelec2	-	--	-
(14)	*B*	A/m	*B_n_*	*B_n_*	Constant	-	*B_n_*	constant	$B_{side1}=B_{side2}$	$B_{side1}=B_{side2}$
	$A\times \vec{n}$	T m	-	-	-	0	-	-	-	-

**Table 4:** Boundary conditions applied to the filler wire and workpiece domains.

			Filler Wire Domain (Solid)			Workpiece Domain (Fluid)				
			Wall	Sym.	Wall	Wall	Sym.	Wall	Wall	Wall
Equation No.	Boundary Variable	Units	*i*	*j*	*k*	*h*	*m*	*n*	*o*	*p*
(15) and (16)	*U*	m/s	-	-	-	-	$U_{n}=0$	0	0	0
	*p*	Pa	-	-	-	Gas drag + *M_a_*	-	-	-	-
(17) and (19)	*T*	K	303	$\frac{\partial T}{\partial n}=0$	-	-	$\frac{\partial T}{\partial n}=0$	303	303	303
	*q*	W/m^2^	-	-	$F_{T}$	$F_{T}$	-	-	-	-
	*h_c_*	W/m^2^K	-	-	-	-	-	20	20	20
(12) and (13)	*E*	V/m	-	-	-	-	-	-	0	0
	*V*	Volt	-	-	-	-	-	Ground	-	-
	*J*	A/m^2^	-	-	-	$J_{Arc}$	-	-	-	-
(14)	*B*	A/m	*-*	*-*	-	*B_n_*	Constant	*B_n_*	-	*B_n_*
	$A\times \vec{n}$	T m	-	-	-	-	-	-	0	-

## Data Availability

Not applicable.
